# *A. muciniphila* Supplementation in Mice during Pregnancy and Lactation Affects the Maternal Intestinal Microenvironment

**DOI:** 10.3390/nu14020390

**Published:** 2022-01-17

**Authors:** Yuli Qi, Leilei Yu, Fengwei Tian, Jianxin Zhao, Hao Zhang, Wei Chen, Qixiao Zhai

**Affiliations:** 1 State Key Laboratory of Food Science and Technology, Jiangnan University, Wuxi 214122, China; qiyuli521@163.com (Y.Q.); edyulei@126.com (L.Y.); fwtian@jiangnan.edu.cn (F.T.); zhaojianxin@jiangnan.edu.cn (J.Z.); zhanghao61@jiangnan.edu.cn (H.Z.); chenwei66@jiangnan.edu.cn (W.C.); 2 School of Food Science and Technology, Jiangnan University, Wuxi 214122, China; 3 National Engineering Research Center for Functional Food, Jiangnan University, Wuxi 214122, China; 4 Wuxi Translational Medicine Research Center, Jiangsu Translational Medicine Research Institute Wuxi Branch, Wuxi 214122, China

**Keywords:** *A. muciniphila*, pregnancy, lactation, intestinal barrier, fecal metabolites

## Abstract

During pregnancy and lactation, considerable factors that affect the maternal microbiome are associated with the advancement of numerous diseases, which can potentially affect offspring health. Probiotics have shown potential for the maintenance of microbiota homeostasis of mothers in this period. The specific objective of this study was to investigate whether the application of *Akkermansia muciniphila* (*A. muciniphila*) during pregnancy and lactation impacts maternal and offspring health. Here we show that dams fed with *A. muciniphila* is safe, enhances the intestinal barrier and alters gut microbiota composition and diversity at the end of lactation, including the significant enrichment of *A. muciniphila* and *Ruminococcus_1* in offspring from probiotic-fed dams. However, compared with the control group, the fecal metabolites of the *A. muciniphila* group only changed slightly. Additionally, *A. muciniphila* supplementation did not significantly increase the abundance of *A. muciniphila* in the fecal microbiota of offspring mice. Compared with the control group, the fecal metabolic profile of three-week-old offspring of mice fed with *A. muciniphila* were significantly changed, containing the D-glutamine and D-glutamate metabolism pathways. These results provided evidence that *A. muciniphila* supplementation in mice during pregnancy and lactation is safe and seemed to have a more beneficial effect on dams. In the future, using probiotics to regulate maternal microbiomes during pregnancy and lactation could be shown to have a more lasting and beneficial effect.

## 1. Introduction

Knowledge on gut microbiota has greatly expanded over the last few years, and growing evidence from various epidemiological studies suggests that allergy, obesity, metabolic syndrome, and other chronic diseases in adulthood are related to the disruption of gut microbiota early in life. A recent study showed that children who were prescribed antibiotics early in life (within the first 2 years) were at increased risk of developing conditions such as asthma in the future [1]. At birth, native microbes quickly and intensively adapt to and take over the gut of a newborn, and these early colonizers can significantly affect gut microbiota development via a “priority effect,” and the taxonomic and functional characteristics of the gut microbiota at birth can persist even after environmental changes [2]. The maternal microbiome during pregnancy or lactation is the dominant force affecting the gut microbiota of the offspring. The maternal microbiome may be transmitted directly to the baby vertically through the gut, vagina, and breast milk [3], or they may indirectly guide the establishment of neonatal microbiota and immune development via their metabolites [4,5]. Population-based cohort studies showed that the alpha diversity of the maternal fecal microbiota during the third trimester of pregnancy can predict the brain and behavioral development of the child at 2 years of age [6]. A new study from an Australian prebirth cohort has suggested a notable correlation between the existence of Prevotella copri in mothers’ gut during pregnancy and the risk of allergic disease in their offspring. Furthermore, increasing the abundance of *P. copri* in the guts of pregnant mothers may help prevent food allergies in children [7]. Oral probiotics have many benefits for human health [8,9,10,11]. Some probiotics or mixed probiotics can be used to interfere with the microbiota of mothers, which may have beneficial effects on the offspring. 

The colon mucus layer is a heavily O-glycosylated mucin layer that is tightly attached to the intestinal epithelium. This layer plays a vital role in preventing the gut microbiota from activating intestinal immune cells, which may lead to inflammation and cancer. The main component of intestinal mucus is MUC2, produced primarily by goblet cells in the distal colon. *A. muciniphila*, a probiotic with great application potential, is common in the gastrointestinal (GI) tract of mammals and can regulate mucin secretion [12]. Studies have presented evidence that *A. muciniphila* supplementation can repair the ethanol-induced disruption of intestinal barrier integrity, increase the thickness of intestinal mucus, and increase the expression of tight junction proteins [13]. Moreover, *A. muciniphila* administration extended crypt length, increased the number of goblet cells, and improved colitis in mice fed a low-cellulose diet [14]. In addition, previous studies have shown that *A. muciniphila* can improve metabolic function as well as prevent high-fat-diet-induced obesity [15] and type I and type II diabetes [16,17,18]. A novel bacterial strain, *A. muciniphila* WB-STR-0001, has been shown to be safe, well-tolerated, and able to help control postprandial glucose in patients with type 2 diabetes [19]. 

*A. muciniphila* is present in the GI tract of 1-month-old infants (at the earliest), whereafter it colonizes steadily within the first year after birth, eventually accounting for approximately 1–4% of the microbial community in healthy adults [20]. Furthermore, *A. muciniphila* has the ability to metabolize oligosaccharides in breast milk, which may contribute to microbiome establishment and immune maturation in the early life of infants [21]. A new study found that betaine intake during lactation increased betaine content in breast milk, resulting in a temporary increase in the ratio of *A. muciniphila* in early life, decreased adiposity, and improved glucose homeostasis in the offspring in adulthood [22]. *A. muciniphila* treatment successfully prevented hippocampus-dependent learning and memory deficits in mice fed high-fat diet early in life [23]. Therefore, increasing the abundance of *A. muciniphila* may be a promising strategy to enhance infant health during early life. 

Many studies have investigated the influence of probiotic supplementation during pregnancy in women with obesity/diabetes. A randomized controlled clinical trial found that probiotic yoghurts, including *Lactobacillus* and *Bifidobacterium*, improved glucose metabolism in overweight and obese pregnant women [24]. In addition, administration of specific probiotic strains has been reported to exert beneficial effects in the offspring, such as preventing allergic disease [25] and infantile colic [26]. However, most studies have focused on Lactobacillus and Bifidobacterium or mixtures of the two strains. Thus, it remains unclear whether *A. muciniphila* has a positive effect on maternal and fetal health. In light of this, this study aimed to investigate the effects of *A. muciniphila* intake during pregnancy and lactation on the productive performance of mothers, as well as to monitor the physiological growth indices, changes in microbiota composition, and metabolites of the mothers and their offspring.

## 2. Materials and Methods

### 2.1. Bacterial Strains and Preparation

*A. muciniphila* MucT (ATCC BAA-835) was obtained from the American Type Culture Collection (ATCC). The strain was cultured as described previously [21]. The bacterial culture medium was brain–heart infusion medium (Qingdao Hope Bio-Technology Company, Qingdao, China) supplemented with hog gastric mucin (0.25% *w*/*v* Type II; Sigma-Aldrich, St. Louis, MO, USA). The bacterial cells were grown under anaerobic conditions at 37 °C for 18 h, and were enriched by centrifugation (6000× *g*, 10 min) and washed with normal saline. Finally, the bacterial sludge was resuspended in normal saline at the required final concentration.

### 2.2. Animals

Male and female C57BL/6 mice (8 weeks of age) were purchased from Charles River Laboratory Animal Technology Co., Ltd. (Beijing, China) and then housed in the Laboratory Animal Center of the Department of Food Science and Technology, Jiangnan University, Wuxi, China. The animals were housed under specific pathogen-free conditions. Male and female mice were randomly selected and mated for 24 h, with a mating ratio of 1:1. After mating (P1), all female mice were individually housed and monitored daily until birth. After observation and recording of the changes in mouse weight and body shape, the female mice confirmed not to be pregnant were again caged with male mice to ensure that there were 10 pregnant female mice in each group. The day of birth was the first day of the offspring (B1). It was guaranteed that each lactating female mouse fed 5–8 pups, which were allowed free access to breast milk. At 3 weeks of age, the pups were weaned, separated from their mother, and housed in populations of five per cage.

### 2.3. Experimental Design

**Figure 1:**
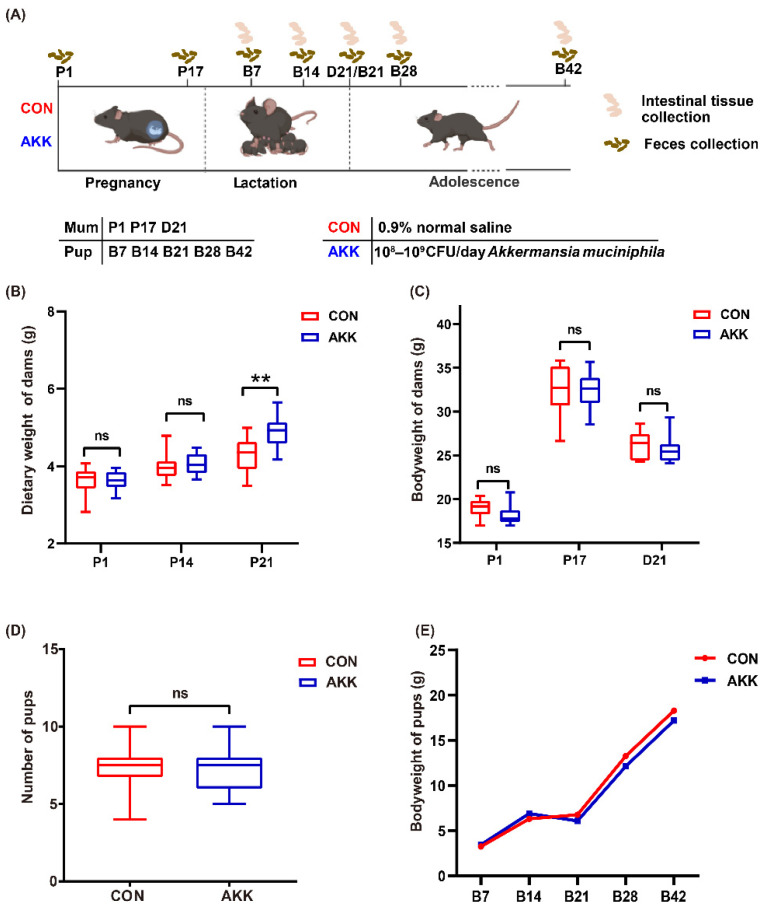
*A. muciniphila* supplementation had no significant effect on the basic physiological indexes of mouse dams and pups. (**A**) Experimental design: Mothers were fed *A. muciniphila* or 0.9% normal saline starting from the first day of pregnancy (P1) to the end of lactation (D21). Starting from postnatal day 7 (B7), pups were sacrificed at regular intervals for sample collection. All mice were fasted and weighed before sacrifice. (**B**) Dietary weight of dams. (**C**) Bodyweight of dams during pregnancy and lactation. (**D**) The number of pups in each group. (**E**) Bodyweight of pups from day 7 to day 42 after birth. ns: no significant difference between the two groups, ** *p* < 0.01 ns: not significant difference.

All animals were randomized into two groups: (1) CON group and (2) AKK group. Each group consisted of 10 mothers and their respective 5–8 pups. Except for 3 days before and after birth, mothers in the AKK group were fed *A. muciniphila* (10^8^–10^9^ CFU/day) by oral gavage for 6 weeks, including 3 weeks of gestation and 3 weeks of lactation. The CON group, i.e., the control group, received normal saline under the same conditions. The samples of mothers and pups collected at different time points are shown in Figure 1A. Maternal and offspring body weight was monitored weekly. Feces from mothers were collected twice during pregnancy (P1, P17) and once on the 21st day of lactation (D21). Feces from pups were collected after birth (B7, B14, B21, B28, and B42). After weaning, all mothers were sacrificed. At each time point (B7, B14, B21, B28 and B42), 8–10 mice from 3–10 different cages in each group were sacrificed. The colon and ileum of each mouse were quickly removed for further analysis. 

### 2.4. Histology

The colon and ileum of mice were excised and fixed in 10% neutral buffered formalin or Carnoy’s fluid. Tissue sections were embedded and stained with hematoxylin and eosin (H and E) or periodic acid-Schiff (PAS), as described previously [27]. All images were captured using a Leica BA410E microscope. Crypt height and the number of goblet cells were quantified by a blinded investigator. 

### 2.5. Total RNA Extraction and qPCR Analysis

Total tissue RNA was extracted from colonic and ileal tissues and was reverse-transcribed into complementary DNA using the HiScript^®^ III RT SuperMix R333-01 for qPCR (+gDNA wiper) kit (Vazyme Biotech Co., Ltd., Nanjing, China). qPCR was performed with SYBR green (iTaq™ Universal SYBR^®^ Green SuperMix; Bio-Rad Laboratories Co., Ltd., Shanghai, China) on a real-time PCR system (CFX384 Real-Time PCR Detection System; Bio-Rad Laboratories Co., Ltd., Shanghai, China), using β-actin as a housekeeping gene. Primer sequences are listed in Supplementary Material Table S1. 

### 2.6. Fecal Sample Collection and Genomic DNA Extraction

Fecal samples from each mouse were collected rapidly in a sterile tube and stored at −80 °C until genomic DNA extraction. Genomic DNA was obtained using the Fast DNA Spin Kit for Feces (MP Biomedicals, Carlsbad, CA, USA). Its quantity and quality were assessed using a NanoPhotometer^®^N50 (Implen, Inc., Westlake Village, CA, USA). The V3–V4 regions of the 16S rRNA gene were amplified using the primers 341F/806R. Amplicons were then purified using a DNA Gel/PCR Purification Miniprep Kit (Biomiga, Hangzhou, China). The purified DNA was added to an equimolar solution and pooled. For library preparation, the DNA samples were sequenced on an Illumina MiSeq PE300 platform (Illumina, San Diego, CA, USA). 

The paired-end sequences were denoised and merged, and chimeras were removed and filtered using the DADA2 plugin. Quality-controlled data were analyzed using the QIIME 2 pipeline [28]. Taxonomic sequence classification was performed using the classifier trained on the V4 515–806 bp regions of the 16S rRNA gene sequences from the Silva rRNA reference database. Analysis of the structure of gut microbiota was performed using the QIIME 2 software and calculated using the R software.

### 2.7. Detection of A. muciniphila by qPCR

The ratio of *A. muciniphila* in stool samples was determined by qPCR, as described previously [29]. Bacterial DNA with a known colony-forming unit value was extracted using a TIANamp Bacteria DNA kit (TIANGEN, Beijing, China), and a standard curve of *A. muciniphila* was established with a range of 10^2^ to 10^10^ cells. Amplification was conducted using a real-time PCR system (CFX384 Real-Time PCR Detection System; Bio-Rad Laboratories). The absolute abundance of *A. muciniphila* in each stool sample was calculated based on the Cq value of the standard curve. All assays were performed twice. Fecal sample DNA was extracted as described in Section 2.6. 

### 2.8. Untargeted Metabolomics Analysis

Fecal metabolites were analyzed as previously described [30,31]. Fecal pellets (60 mg) from mothers (P1, P17, and D21) and pups (B21 and B42) were collected directly into individual 1.5-mL centrifuge tubes and homogenized with 600 μL of methanol/acetonitrile/deionized water mixture (2:1:1, *v*/*v*/*v*, precooled to −20 °C), and then centrifuged at 4 °C (12,000× *g*, 10 min). The supernatant (400 μL) was concentrated, dried for 2 h under vacuum, and reconstituted with 200 μL of methanol/deionized water (4:1, *v*/*v*). Next, the samples were vortexed and centrifuged as described above. Using a 0.22-μm membrane filter, the supernatant was filtered into a sample bottle for further analysis. To prepare quality control (QC) samples, 2 μL of each sample was placed in the same tube and mixed. Methanol (80%) was used as the blank control sample.

Metabolites were detected using a Dionex Ultimate-3000 ultrahigh-performance liquid chromatography (UPLC) system (Thermo Fisher Scientific, Waltham, MA, USA) coupled with a high-resolution Q-ExactiveOrbitrap mass spectrometer system in the full scan mode (Thermo Fisher Scientific, Waltham, MA, USA). An Acquity HSS T3 column (2.1 mm × 100 mm, 1.8 µm; Waters, MA, USA) was used for UPLC separation, at a flow rate of 0.3 mL/min and injection volume of 2 µL. The chromatographic and mass spectrometric conditions are listed in Table S2. 

Metabolomics data were analyzed using the Compound Discoverer 3.2 software (Thermo Fisher Scientific) as described previously [31]. The orthogonal partial least-squares discriminant analysis (OPLS-DA) model was used to identify the metabolic features that represented the difference between the two groups. Metabolic features with variable importance in projection (VIP, VIP > 1, and *p* < 0.05) were reserved as potential differential biomarkers. Metabolic pathway analysis based on the identified metabolites was conducted using MetaboAnalyst 5.0.

### 2.9. Statistical Analysis

The GraphPad Prism 8 software (version 8.2.1 Windows version, GraphPad Software, San Diego, CA, USA) and charting tools in Hiplot (https://hiplot.com.cn, accessed on 8 October 2021), a comprehensive web platform for scientific data visualization, were used for data processing. Statistical comparisons were conducted using one-way or two-way analysis, followed by Tukey’s test or unpaired t-test. The data for each treatment are shown as the mean ± SEM. Statistical significance was considered at * *p* < 0.05, ** *p* < 0.01, and *** *p* < 0.001. “ns” means no significant difference between the two groups.

## 3. Results

### 3.1. Effects of A. muciniphila Supplementation on the Basic Physiological Indexes of Mothers and Pups

There was no significant reduction in dietary weight and body weight between *A. muciniphila*-fed and placebo-fed dams (Figure 1B,C). The amount of food intake was measured weekly during pregnancy, and our analysis showed that the AKK group had significantly increased food intake before birth (P21) than the CON group (4.29 ± 0.44 vs. 4.89 ± 0.49 for the CON group vs. the AKK group, respectively, *p* = 0.002) (Figure 1B). As the numbers of pups per litter were not always the same, and these pups had started ingesting solid foods in the later lactation period, the amount of maternal food intake during lactation was not recorded. By the time before birth (P17), dams in both groups weighed approximately 32 g. As the lactation period ended, maternal weight decreased (Figure 1C). In addition, *A. muciniphila* supplementation had no adverse effects on gestational outcomes. All female mice delivered naturally, and the number of offspring was not affected by *A. muciniphila* supplementation (Figure 1D). Over time, the pups grew normally and gained weight, with no significant difference between the two groups at any time point (Figure 1E).

### 3.2. Effects of A. muciniphila Supplementation on the Intestinal Barrier of Mothers

**Figure 2:**
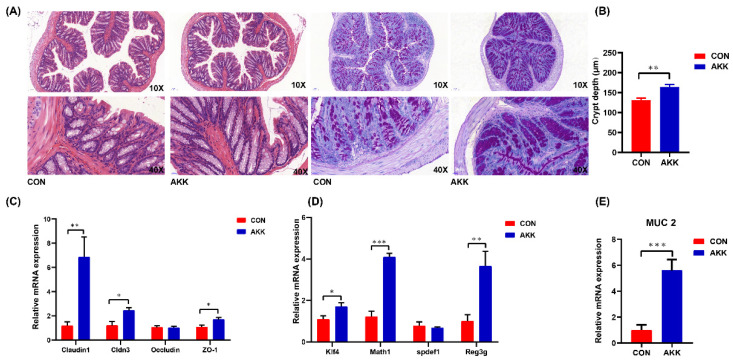
Effects of *A. muciniphila* supplementation during pregnancy and lactation on the intestinal barrier of mouse dams. (**A**) Colonic tissues of mothers at the end of lactation (D21) were observed by hematoxylin-eosin (**left**) and periodic acid-Schiff staining (**right**). (**B**) Crypt depth of at least 100 villi or crypts per mice was detected. (**C**,**D**) mRNA expression of genes associated with the intestinal barrier. (**E**) mRNA expression of mucin 2 (MUC2). * *p* < 0.05, ** *p* < 0.01, *** *p* < 0.001.

*A. muciniphila* supplementation during pregnancy and lactation markedly enhanced the intestinal barrier of mothers (D21). Compared with those in the CON group, the number of goblet cells and crypt depth were significantly increased in the AKK group (Figure 2A,B). Analysis of tight junction (TJ) mRNA expression indicated that *A. muciniphila*-fed mothers had higher mRNA expression levels of claudin 1, claudin 3, and ZO 1 than mothers in the CON group. Furthermore, occludin mRNA expression was not affected (Figure 2C), whereas mucin 2 (MUC2) mRNA expression was increased (Figure 2E). The mRNA expression levels of genes related to goblet cells, including Krüppel-like factor 4 (Klf4) and Math1, showed similar significant changes. However, Spedf mRNA expression was not significantly altered in the AKK group compared with that in the CON group (Figure 2D). Reg3g gene expression in the AKK group was significantly upregulated compared with that in the CON group (Figure 2D). Overall, these results suggest that *A. muciniphila* supplementation during pregnancy and lactation enhances the intestinal barrier.

### 3.3. Maternal A. muciniphila Supplementation Did Not Affect the Intestinal Barrier of Offspring

**Figure 3:**
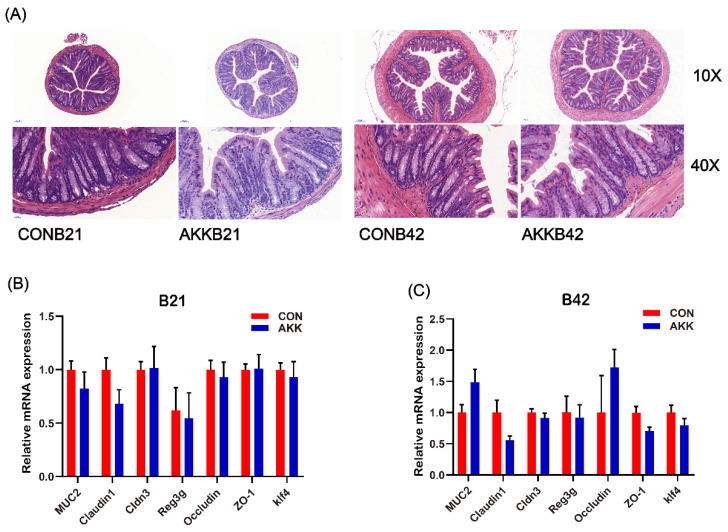
Effects of *A. muciniphila* supplementation during pregnancy and lactation on the intestinal barrier of mouse offspring. (**A**) Representative image of hematoxylin–eosin-stained colon tissue of offspring on days 21 and 42 (B21 and B42) after birth. The mRNA expression of genes related to the intestinal barrier of offspring at B21 (**B**) and B42 (**C**) in different groups.

To investigate whether *A. muciniphila* supplementation in mothers affects the intestinal barrier of their offspring, offspring mice were observed for up to 42 days. In the CON group, the depth of intestinal crypts and the mRNA expression of genes related to the intestinal barrier increased rapidly with time, and the time of weaning (B21) was a key turning point (Figure S1). Furthermore, comparison of the colon sections of offspring on days 21 and 42 (B21 and B42) after birth showed that *A. muciniphila* supplementation did not affect the development of intestinal tissue structure (Figure 3A). No difference was observed in the expression of TJ genes and the number of goblet cells in pups, and MUC2 expression showed the same trend in the AKK group at B21 and B42 (Figure 3B). Comparisons between the two groups at other time points showed the same conclusion (Figure S2A,B).

### 3.4. Effects of A. muciniphila Supplementation on the Gut Microbiota Composition of Mothers

**Figure 4:**
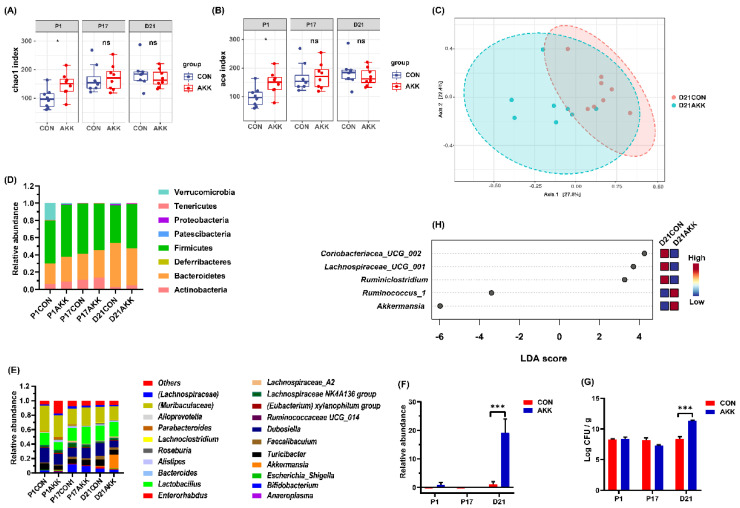
*A. muciniphila* supplementation results in favorable alterations in maternal gut microbiota. (**A**) The Chao1 index of mother mice. (**B**) The ACE index of mother mice. (**C**) PCoA indicating the β-diversity of gut microbiota in mother mice. Major changes in bacterial phyla (**D**) and genera (**E**) in mother mice. (**F**) *A. muciniphila* relative abundance in maternal feces. (**G**) *A. muciniphila* absolute concentration in maternal feces. (**H**) LEfSe analysis of maternal gut microbiota at D21. * *p* < 0.05, *** *p* < 0.001, ns: not significant difference.

The gut microbiota compositions of mothers at P1, P17, and D21 were analyzed. At the beginning of pregnancy (P1), namely the first day of *A. muciniphila* supplementation, the community richness index (Chao1 and ACE indices) of pregnant mice was significantly increased (Figure 4A,B), but the community diversity index showed no significant change (Figure S3). As pregnancy progressed, the gut microbiota of the mothers tended to be stable. The AKK group did not display any significant differences in richness and diversity indices. Dramatic differences in the β-diversity of gut microbiota were only observed at D21 (Figure 4C; Bray–Curtis index, *p* < 0.006). As the mice underwent pregnancy and lactation, the structure of their gut microbiota changed (Figure 4D,E). At the phylum level, the abundance of Actinobacteria first increased and then decreased (Figure 4D; 0.05 vs. 0.13 vs. 0.09, P1 vs. P17, *p* = 0.014; P17 vs. D21 *p* = 0.85, in the CON group), whereas the relative abundance of Bacteroidetes gradually decreased (Figure 4D; 0.43 vs. 0.32 vs. 0.29, P1 vs. P17, *p* = 0.13; P17 vs. D21 *p* = 0.96, in the CON group). *A. muciniphila* supplementation shifted the composition of gut microbiota and increased the relative abundance and absolute concentration of *A. muciniphila* in maternal feces at D21 (Figure 4F,G; *p* < 0.001, *p* < 0.001). Furthermore, supplementation with probiotics increased the relative abundance of *Ruminococcus_1*, but decreased the ratios of *Coriobacteriaceae* UCG-002, *Lachnospiraceae* UCG-001, and *Ruminiclostridium* at D21 (Figure 4H; *p* < 0.05).

### 3.5. Effects of A. muciniphila Supplementation on the Gut Microbiota Composition of Offspring

**Figure 5:**
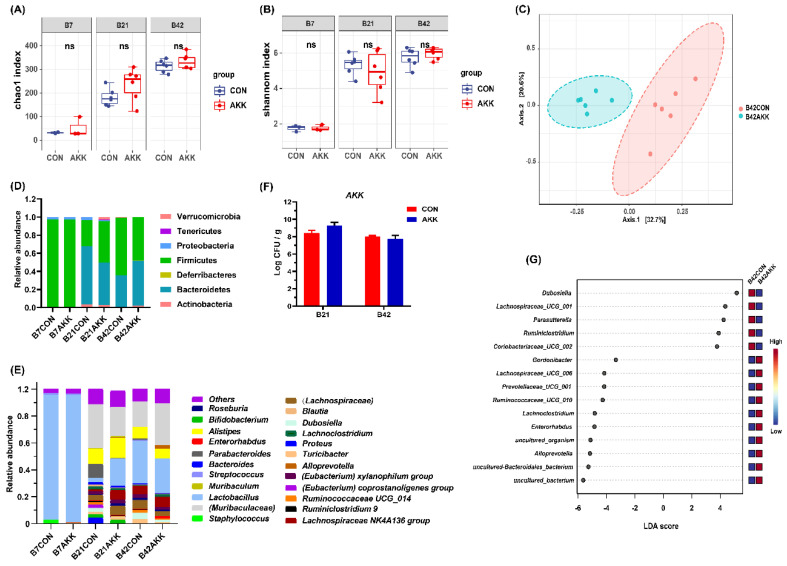
*A. muciniphila* supplementation results in favorable alterations in the gut microbiota of pups. (**A**) The Chao1 index of pups. (**B**) The Shannon index of pups. (**C**) PCoA describing the β-diversity clustering of the gut microbiota of pups. Major changes in bacterial phyla (**D**) and genera (**E**) in pups. (**F**) *A. muciniphila* absolute concentration in maternal feces. (**G**) LEfSe analysis of maternal gut microbiota at D21. ns: not significant difference.

To determine whether *A. muciniphila* supplementation to mothers during pregnancy and lactation also affected the composition of gut microbiota of their offspring, stool analysis was conducted by 16S rRNA gene sequencing. We focused on three time points: the seventh day of birth (B7), day after weaning (B21), and post-pubertal phase (B42). There was no significant difference in the α-diversity of gut microbiota between the offspring of the two groups at any time point (Figure 5A). Furthermore, β-diversity analysis results showed that microbial signatures were distinctly clustered in different group at B42 (Figure 5B; Bray-Curtis Index, *p* < 0.03), but not at the other time points (Figure S4a,b). The composition of intestinal microbiota of the offspring of *A. muciniphila*-fed mice was not significantly different from that of the CON group on the 21st day after birth, but its composition was similar to that on day 42 (Figure 5D,E). The relative abundance of *A. muciniphila* in the offspring of the AKK group at day 21 was higher than that in the CON group, but the difference was not significant (Figure S4c). The results of qPCR analysis for *A. muciniphila* in pup fecal samples were similar to the sequencing results, indicating that there was no significant difference in the abundance of *A. muciniphila* in the feces of 21- and 42-day-old pups between the two groups (Figure 5F). LEfSe analysis showed that at B42, the offspring of mothers receiving *A. muciniphila* had decreased abundance of *Dubosiella*, *Lachnospiraceae*_UCG_001, and *Parasutterella*, and increased abundance of *Gordonibacter*, *Lachnospiraceae*_UCG_006, and *Prevotellaceae*_UCG_001, compared with the offspring of the CON group (Figure 5G; *p* < 0.05).

### 3.6. Fecal Metabolites of Mothers and Pups Are Modified by A. muciniphila Supplementation

**Figure 6:**
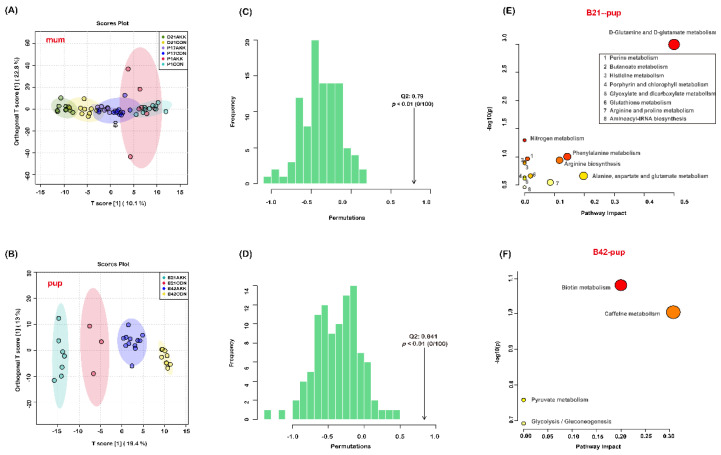
Effects of *A. muciniphila* supplementation during pregnancy and lactation on the fecal metabolites of mothers and pups. OPLS-DA score plots of fecal metabolites of mothers (**A**) and pups (**B**) at different time points. The distribution of test statistic (Q2) and *p* value for permutation test of (**C**,**D**). Pathway analyzes of the differential metabolites in pups at B21 (**E**) B42 (**F**).

To further analyze the effects of *A. muciniphila* supplementation on the intestinal metabolites of mothers and pups, untargeted metabolomics analysis was conducted to identify changes in metabolites in mouse feces. After data filtering using the CD software, 448 and 449 different metabolites under the POS and NEG ion modes were successfully identified in the feces of dams and pups, respectively (Tables S3 and S4). OPLS-DA was applied to discriminate the differential features in the stool samples of mothers and pups. The results showed a segregation between mothers in the CON and AKK groups only at D21 (Figure 6A). However, significant clustering was observed among pups at different time points (Figure 6B). Permutation test further validated the good predictability of OPLS-DA (Figure 6C,D). Furthermore, univariate analysis was performed to identify important differential metabolites between the two groups. A total of one, six, and nine metabolites were identified as differential biomarkers (log2(FC) > 1 and *p* < 0.05) in mothers of the AKK group at P1, P17, and D21, respectively, and 19 and 23 metabolites were identified as differential biomarkers (log2(FC) > 1, *p* < 0.05) in pups of the AKK group at B21 and B42, compared with the CON group at the corresponding time points (Table 1). Metabolic pathway analysis using MetaboAnalyst showed that the metabolites with significant changes at B21 are involved in 13 metabolic pathways, and that *A. muciniphila* had the greatest effects on the D-glutamine and D-glutamate metabolism pathways (impact = 0.5), which were significant (*p* < 0.05) (Figure 6E). At B42, the altered metabolites were related to biotin metabolism, caffeine metabolism, pyruvate metabolism, and glycolysis/gluconeogenesis (*p* > 0.05) (Figure 6F). The differential metabolites in the other groups could not be associated with relevant pathways. 

## 4. Discussion

Numerous perinatal factors, including diet, antibiotics, stress, different labor types, and feeding methods, have been reported to affect maternal gut microbiota during pregnancy and lactation, affecting offspring immune development and health status in later life [32,33]. Several mechanisms of maternal bacteria in influencing offspring health have been identified. These include mother-to-infant vertical microbial transmission [34], exposure of the fetoplacental unit to bacteria [35], passing of microbial metabolites to the fetus through the umbilical cord [36], and immunoglobulin-related transplacental transport of gut bacterial components. Therefore, pregnancy and lactation can be considered the window period affecting the microbiome establishment and lifelong health of the offspring. This study investigated the effect of *A. muciniphila* supplementation on mothers and pups.

We observed that *A. muciniphila* supplementation increased maternal food intake before birth (P21) but did not affect maternal bodyweight and pregnancy outcome. Constant intake of *A. muciniphila* resulted in increased mucus secretion, intestinal goblet cell number, and expression of genes associated with the intestinal barrier (e.g., Claudin-1, Cldn3, and ZO-1) in mother mice at the end of lactation. These effects were accompanied by a change in the bacterial community structure and a transient increase in the ratio of *A. muciniphila* in maternal gut microbiota. Moreover, *A. muciniphila* intake slightly changed maternal fecal metabolites during pregnancy and lactation. However, *A. muciniphila* supplementation during pregnancy and lactation did not significantly affect the growth and intestinal barrier of offspring mice, but disturbed the microbial community structure and metabolism, as indicated by increased abundance of *Dubosiella*, *Lachnospiraceae*_UCG_001, and *Parasutterella*, as well as decreased abundance of *Gordonibacter*, *Lachnospiraceae*_UCG_006, and *Prevotellaceae*_UCG_001 at B42.

Our results suggest that *A. muciniphila* supplementation to pregnant and lactating mice is safe and does not lead to any side effects on mothers or pups. Similarly, supplementation of Lactobacillus fermentum CECT5716 did not modify the body weight, body mass index, and Lee index of dams and pups [37]. Most clinical trials also support the safety of probiotics supplementation before, during, and after pregnancy [38,39,40]. *A. muciniphila* is one of the most common microbes in the mammalian gut. At present, *A. muciniphila* WB-STR-0001 has been successfully applied as a probiotic medical food to improve the symptoms of type 2 diabetes [41]. In summary, the results of this study add to the evidence that supplementation of the probiotic strain *A. muciniphila* at appropriate doses during pregnancy and lactation is safe.

As a physical and immune defense, the intestinal barrier plays an important role in combating microbes, food antigens, and environmental toxins. As an intestinal commensal bacterium, *A. muciniphila* exerts its beneficial effects, at least in part, by improving the intestinal barrier function. In adult obese mice, daily oral administration of *A. muciniphila* for approximately 1 month increased the number of ileal goblet cells and the expression of intestinal barrier markers, thereby reducing systemic inflammation and improving metabolic health [42,43,44]. *A. muciniphila* has been implicated in the control of host mucus turnover [15]. The results of 16 sRNA sequencing and qPCR analyses showed that the abundance of *A. muciniphila* in female mice at D21 increased significantly after continuous *A. muciniphila* supplementation during pregnancy and lactation. Recently, several studies have indicated that elevation in the abundance of *A. muciniphila* cells leads to increased number of goblet cells [45]. Similarly, our data showed that 6 weeks of *A. muciniphila* supplementation increased the number of goblet cells and the expression of MUC2 in mother mice. MUC2 is a major component of the intestinal mucus that is essential to protect the gastrointestinal tract.

TJ proteins are also important for maintaining the integrity of the intestinal barrier [46]. Numerous evidence has shown that decreased expression and translocation of TJ proteins might cause intestinal damage. In the current study, *A. muciniphila* significantly increased the mRNA expression of claudin-1, Cldn3, and ZO-1, but not that of occludin in mother mice. Furthermore, *A. muciniphila* administration during pregnancy and lactation led to the upregulation of the regenerating family member 3 gamma (Reg3g) gene, which is essential for defense against the colonization of Gram-positive bacteria, distribution of intestinal mucus, and immune response to pathogens [30]. Thus, *A. muciniphila* administration enhanced the gut barrier function of mother mice.

Our results showed that the intestinal microbiota diversity and richness in dams were disturbed only at the beginning of *A. muciniphila* supplementation and reached a stable state in the later period. At D21, *A. muciniphila* supplementation not only increased the abundance of *A. muciniphila* but also affected the relative abundance of other bacteria (Figure 4). A previous study on the effect of liraglutide in improving nonalcoholic fatty liver disease in mice showed a similar relationship between the abundances of *A. muciniphila* and these bacteria; liraglutide significantly increased the ratio of *A. muciniphila* whilst decreasing the abundance of *Bacteroides*, *Lachnospiraceae_UCG-001*, *Lachnospiraceae*_NK4A136_group, *Klebsiella*, *Anaerotruncus*, and *Ruminiclostridium* [47]. A controlled clinical trial showed that orange juice increased the abundance of *Lactobacillus* spp., *A. muciniphila* spp., and *Ruminococcus* spp. and simultaneously improved glycemia and lipid profiles [48]. A higher abundance of *Ruminococcus_1* was associated with improved polycystic ovary syndrome [49]. Thus, *A. muciniphila* supplementation may exert beneficial changes in the intestinal microbiota structure of mother mice. Moreover, there were significantly different clusters of intestinal metabolites in mother mice at day D21. 

In contrast to the results in maternal mice, *A. muciniphila* supplementation caused minor changes in the gut microbiota of offspring mice, without a significant increase in the abundance of *A. muciniphila*. As there is a trace amount of oxygen in the neonatal gut, the first colonizers in the intestine are mainly the typical facultative anaerobes *Lactobacillaceae* and *Enterobacteriaceae*. With the consumption of oxygen, obligate anaerobes, including *Bifidobacterium*, *Bacteroides*, and *Clostridium*, gradually colonize the intestines of infants [50]. *A. muciniphila* is a strictly anaerobic bacterium, making it difficult to pass from mother to infant through breastfeeding. In a clinical study, mothers were administered probiotic milk containing LGG, Bb-12, and La-5 from 36 weeks of gestation to 3 months postpartum with breastfeeding, but only LGG led to a transient colonization in the infants at 10 days and 3 months of age [51]. This result proves that different probiotic strains have different vertical transmission abilities from mother to child. It is possible that the strain used in this study cannot spread vertically. This may be because *A. muciniphila* did not significantly affect the intestinal microbiome of offspring; thus, there was no significant difference in intestinal barrier development between pups in the two groups. Interestingly, in our study, PCoA results revealed that the intestinal microbiota of the two groups of pups showed significantly different clustering on day 42 after birth, and the relative abundance of some bacterial species was changed. This suggests that longer monitoring of mice may be needed, and the effects of *A. muciniphila* on offspring require a longer time to manifest. 

Microbial molecules (such as LPS) and metabolites (such as retinoids, short-chain fatty acids, and secondary bile acids) in the gut of pregnant mothers can penetrate the placental barrier and directly affect the fetus [36]. The maternal microbiome regulates maternal metabolites and fetal brain metabolites, such as trimethylamine-N-oxide and imidazole propionate, promoting the growth of embryonic thalamic axons in vitro [52]. With this in mind, we also analyzed the fecal metabolites of mothers and pups. Our findings showed that probiotic supplementation slightly affected the fecal metabolism of mother mice. However, the fecal metabolic profile of the offspring mice changed more dramatically in comparison. The D-glutamine and D-glutamate metabolism pathways changed significantly in the offspring at B21 (impact = 0.5). Azagra-Boronat et al. revealed that probiotic supplementation in pregnant and lactating mothers might affect their offspring by inducing a lower proportion of palmitic acid and higher proportions of linoleic and α-linolenic acids in breast milk [37,53]. It is possible that *A. muciniphila* supplementation affects metabolites in breast milk.

Overall, *A. muciniphila* supplementation during pregnancy and lactation appeared to have a greater beneficial effect on the mothers than on the offspring, such as strengthening the intestinal barrier and improving the structure of the gut microbiota. *A. muciniphila* supplementation during pregnancy and lactation in mice did not directly increase the abundance of this bacteria in the offspring’s intestines. Yet, it is true that increasing the abundance of *A. muciniphila* in the intestine in the early stages of life may have some physiological significance. Genistein supplementation to mother mice fed a high-fat diet also induced a significant enrichment of the relative abundance of *A. muciniphila* in the intestines of their offspring, while improving glucose homeostasis and insulin sensitivity in offspring [54]. Therefore, it is necessary to further explore the significance of increasing the abundance of *A. muciniphila* and how to increase it in early life. Although this study showed that *A. muciniphila* was safe to be taken during pregnancy, our results were limited to animals; hence, further clinical evaluation is required to examine the potential safety risks for mothers and infants.

## 5. Conclusions

In summary, *A. muciniphila* is safe for use in mice during pregnancy and breastfeeding. Administration of *A. muciniphila* significantly strengthened the intestinal barrier of mother mice by increasing the colonic mucosa crypt depth, number of goblet cells, and mRNA expression of MUC2 and Reg3g, which are TJ protein-related genes. These improvements might be correlated with changes in the diversity and composition of the intestinal microbiota of dams, increased relative abundance of *A. muciniphila* and Ruminococcus_1, as well as decreased proportions of *Coriobacteriaceae* UCG-002, *Lachnospiraceae* UCG-001, and *Ruminiclostridium* in the maternal gut. Fecal metabolite analysis of dams revealed no significant results. Additionally, *A. muciniphila* supplementation caused only small disturbances in the gut microbiota and did not significantly increase the abundance of *A. muciniphila* in the offspring. Compared to that in the CON group, the fecal metabolic profile of offspring in the AKK group was significantly changed at B21. Therefore, probiotic intervention during pregnancy and lactation may improve gut microbial composition and promote health in mothers. It may be possible to design some diet-related substances for dams that could increase *A. muciniphila* abundance and provide long-lasting health benefits in offspring mice.

## Figures and Tables

**Table 1:** Effects of *A. muciniphila* on fecal metabolomics.

Group		Metabolites	Change ^1^	log2(FC)	−log10(*p*)
mum	P1	2,4-quinolinediol	−	5.0125	2.5454
	P17	quercetin	−	3.0601	1.7229
		2-(3,4-dihydroxyphenyl)-5,7-dihydroxy-3,4-dihydro-2H-1-benzopyran-4-one	−	2.6156	2.4021
		N-({(1S,4S,6S)-4-[2-(4-Acetyl-1-piperazinyl)-2-oxoethyl]-6-isopropyl-3-methyl-2-cyclohexen-1-yl}methyl)-3-fluorobenzamide		−1.2058	2.0271
		5(Z),8(Z),11(Z)-eicosatrienoic acid methyl ester	−	1.4851	1.3538
		3-amino-2-phenyl-2H-pyrazolo[4,3-c]pyridine-4,6-diol	−	1.4208	1.53
		2,4-quinolinediol	−	1.3326	1.6166
	D21	chalconaringenin	−	2.2558	4.4939
		chrysin	−	2.2671	3.9032
		2-(3,4-dihydroxyphenyl)-5,7-dihydroxy-3,4-dihydro-2H-1-benzopyran-4-one	−	4.0824	3.7509
		kaempferol	−	4.2064	3.6954
		apigenin	−	1.9089	2.7149
		quercetin	−	3.0535	2.0839
		3-methoxy-5,7,3’,4’-tetrahydroxy-flavone	−	1.2944	2.0068
		1-(3-hydroxy-3-methylpent-4-en-1-yl)-2,5,5,8a-tetramethyl-decahydronaphthalen-2-ol	+	−1.6698	1.5552
		N-({(1S,4S,6S)-4-[2-(4-Acetyl-1-piperazinyl)-2-oxoethyl]-6-isopropyl-3-methyl-2-cyclohexen-1-yl}methyl)-3-fluorobenzamide	+	−1.4211	1.499
pup	B21	ferulic acid	−	1.3433	2.3336
		2-deoxyadenosine	−	1.7339	2.1811
		1,2,3-propanetricarboxylic acid	+	−3.4132	1.9936
		dihomo-γ-linolenic acid ethyl ester	+	−1.1179	1.9883
		4-dodecylbenzenesulfonic acid	+	−1.1009	1.9561
		N-ethylglycine	+	−1.9219	1.9212
		adenine	−	1.3701	1.8602
		trigonelline	−	1.8588	1.7411
		apigenin	−	1.1448	1.6694
		13Z,16Z-docosadienoic Acid	+	−1.3788	1.5585
		D-pyroglutamic acid	+	−1.0427	1.4999
		L-glutamic acid	+	−1.6019	1.4835
		isobutyric acid	−	1.6023	1.4738
		stachydrine	+	−1.4942	1.433
		phenylacetaldehyde	−	1.2711	1.3859
		1-[4-hydroxy-5-(hydroxymethyl)tetrahydrofuran-2-yl]pyrimidine-2,4(1H,3H)-dione	−	1.0677	1.3794
		acamprosate	+	−5.1411	1.354
		L-carnitine	+	−1.4233	1.354
		(1S,19R)-8,10-dioxa-4,17-diazaheptacyclo[15.4.3.01,18 04,19 05,13 07,11 014,19]tetracosa-5(13),6,11,22-tetraen-3-one	+	−1.0226	1.3446
	B42	acamprosate	−	4.3948	6.7137
		NP-003553	−	1.262	5.2985
		NP-015980	+	−1.8399	5.1508
		L-lactic acid	−	1.1072	4.1074
		methyl indole-3-acetate	−	1.3097	3.0721
		tridecylic acid	−	2.6194	2.7511
		ursolic acid	+	−1.0904	2.7069
		1-methylxanthine	+	−1.9701	2.4263
		1-benzyl-3-[(1S,4S)-4-{3,5-dimethyl-1-[4-(2-methyl-2-propanyl)phenyl]-1H-pyrazol-4-yl}-2-cyclopenten-1-yl]urea	+	−1.7189	2.3518
		N-ethylglycine	−	1.0197	2.3183
		3-methylxanthine	+	−1.4299	1.9015
		indole-3-lactic acid	+	−1.2901	1.8742
		5-[({(2R,4S,5R)-5-[1-methyl-3-(2-thienyl)-1H-pyrazol-5-yl]-1-azabicyclo[2.2.2]oct-2-yl}methyl)amino]-5-oxopentanoic acid	+	−3.698	1.8261
		salicylic acid	+	−1.6365	1.7769
		biotin	+	−1.0759	1.7525
		4-(methylthio)-6-phenyl-2-(3-pyridyl)pyrimidine-5-carbonitrile	+	−1.6808	1.6331
		taurochenodeoxycholic Acid _sodium salt_	+	−1.1365	1.5416
		(4aS,5R,6S,8aS)-5-[(3E)-5-methoxy-3-methyl-5-oxopent-3-en-1-yl]-5,6,8a-trimethyl-3,4,4a,5,6,7,8,8a-octahydronaphthalene-1-carboxylic acid	+	−1.2552	1.5266
		4-fluoro-N-{[(1S,4S,6S)-6-isopropyl-3-methyl-4-(2-{[2-(4-morpholinyl)ethyl]amino}-2-oxoethyl)-2-cyclohexen-1-yl]methyl}benzamide	+	−1.0308	1.4497
		1,2,3-propanetricarboxylic acid	−	1.0805	1.4491
		cytosine	−	1.0388	1.4034
		tropine	−	1.8667	1.3217
		N-desmethyltramadol	+	−1.8015	1.3146

^1^ “−”: Compared with the CON group, the abundance of this metabolite at this time point in the AKK group is lower, and “+” means higher.

## Data Availability

Not applicable.

## Supplementary Materials

The following supporting information can be downloaded at: https://www.mdpi.com/article/10.3390/nu14020390/s1, Table S1: Primer sequences for qPCR analyses, Table S2: Instrument conditions for the analysis of metabolomics samples, Table S3: All fecal metabolites in mums, Table S4: All fecal metabolites in pups, Figure S1: The mRNA expression of genes related to the intestinal barrier of mouse offspring in the CON group, Figure S2: Effects of *A. muciniphila* supplementation during pregnancy and lactation on the intestinal barrier of mouse offspring, Figure S3: *A. muciniphila* supplementation results in favorable alterations in maternal gut microbiota, Figure S4: *A. muciniphila* supplementation results in favorable alterations in the gut microbiota of pups.
