# Supplementary material for: A. muciniphila Supplementation in Mice during Pregnancy and Lactation Affects the Maternal Intestinal Microenvironment
Source: Nutrients. 2022 Jan 17;14(2):390. doi: 10.3390/nu14020390 (PMC8779157; doi:10.3390/nu14020390)
Supplement: Supplementary file 1 [file nutrients-14-00390-s001.zip › Supplementary material.docx]

Table S1. Primer sequences for qPCR analyses

| **Gene** | **Forward** | **Reverse** |
| --- | --- | --- |
| MUC 2 | 5’-CCTTAGCCAAGGGCTCGGAA-3’ | 5’-GGCCCGAGAGTAGACCTTGG-3’ |
| Math1 | 5’-GCCTTGCCGGACTCGCTTCTC-3’ | 5’-TCTGTGCCATCATCGCTGTTAGGG-3’ |
| Spedf | 5’-CCGGTTGCCTGCTACTGTTC-3’ | 5’-GCCCATTGCTCCTGATGCT-3’ |
| claudin1 | 5’-GATGTGGATGGCTGTCATTG-3’ | 5’-CCTGGCCAAATTCATACCTG-3’ |
| claudin 3 | 5’-TCATCGTGGTGTCCATCCTGCT-3’ | 5’-AGAGCCGCCAACAGGAAAAGCA-3’ |
| ZO 1 | 5’-CTTCTCTTGCTGGCCCTAAAC-3’ | 5’-TGGCTTCACTTGAGGTTTCTG-3’ |
| occludin | 5’-CACACTTGCTTGGGACAGAG-3’ | 5’-TAGCCATAGCCTCCATAGCC-3’ |
| Klf4 | 5’-GTAGTGCCTGGTCAGTTCATC-3’ | 5’-AACCTATACCAAGAGTTCTCATCTC-3’ |
| Reg3g | 5’-TTCCTGTCCTCCATGATCAAA-3’ | 5’-CATCCACCTCTGTTGGGTTC-3’ |
| β-actin | 5’-GGCTGTATTCCCCTCCATCG-3’ | 5’-CCAGTTGGTAACAATGCCATGT-3’ |
| *A. muciniphila* | 5’-CAGCACGTGAAGGTGGGGAC-3’ | 5’- CCTTGCGGTTGGCTTCAGAT-3’ |

Table S2. Instrument conditions for the analysis of metabolomics samples

| **Instrument conditions** |  | | | |
| --- | --- | --- | --- | --- |
| Spectrum Column | ACQUITY UPLC HSS T3 (2.1×100 mm, 1.8μm) | | | |
|  | Column temperature: 35 ℃ | | | |
| Mobile Phase | For Positive | | | |
|  | A: 0.1% Formic acid in water | | | |
|  | B: 100% Methanol | | | |
|  | For Negative | | | |
|  | A: 100% Water | | | |
|  | B: 100% Methanol | | | |
| Gradient Profile | Time (min) | Percentage B (%) | | Flow rate (mL/min) |
|  | 0.00 | 2.0 | | 0.30 |
|  | 1.00 | 2.0 | | 0.30 |
|  | 10.00 | 98.0 | | 0.30 |
|  | 12.00 | 98.0 | | 0.30 |
|  | 12.10 | 2.0 | | 0.30 |
|  | 15.00 | 2.0 | | 0.30 |
| Injection Volume | 2 μL | | | |
| Mass Parameters | Ion Source | | Electrospray ionization | |
|  | Ionspray voltage | | 3.8 kV | |
|  | Capillary temperature | | 320 ℃ | |
|  | Sheath gas flow velocity | | 35 arb | |
|  | Curtain gas flow velocity | | 15 arb | |
|  | Curtain gas temperature | | 320 ℃ | |
|  | FramsScan | | Full-scan MS/dd-MS2 | |
|  | **Full MS** | |  | |
|  | Resolution | | 70,000 | |
|  | AGC target | | 1e6 | |
|  | Maximum IT | | 100 ms | |
|  | Scan range | | 70 t0 1050 m/z | |
|  | **dd-MS2/dd-SIM** | |  | |
|  | Resolution | | 17,500 | |
|  | AGC target | | 5e4 | |
|  | Maximum IT | | 50 ms | |
|  | Nnormalized collision energy | | 20、40、60 eV | |
|  | Dynamic exclusion | | 10 s | |
|  | TOP N | | 7 | |

**Table S3.** All fecal metabolites in mums. The number is the normalized peak area of the metabolite; The first row is the compound name, and the second line is the sample name.

**Table S4.** All fecal metabolites in pups. The number is the normalized peak area of the metabolite; The first row is the compound name, and the second line is the sample name.


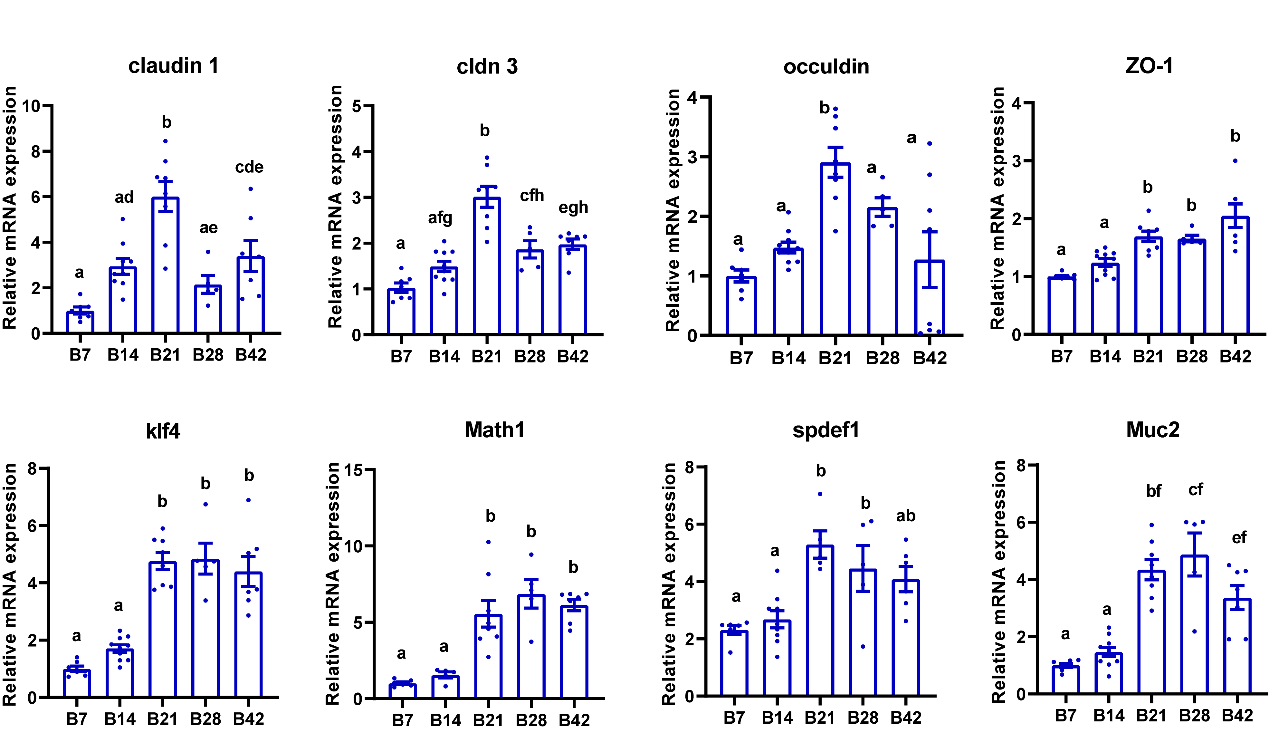


**Figure S1.** The mRNA expression of genes related to the intestinal barrier of mouse offspring in the CON group at B7, B14, B21, B28 and B42. The appearance of the same letter means that there is no marked difference among the groups under the condition of *p* > 0.05; otherwise, there is significant difference, *p* < 0.05.


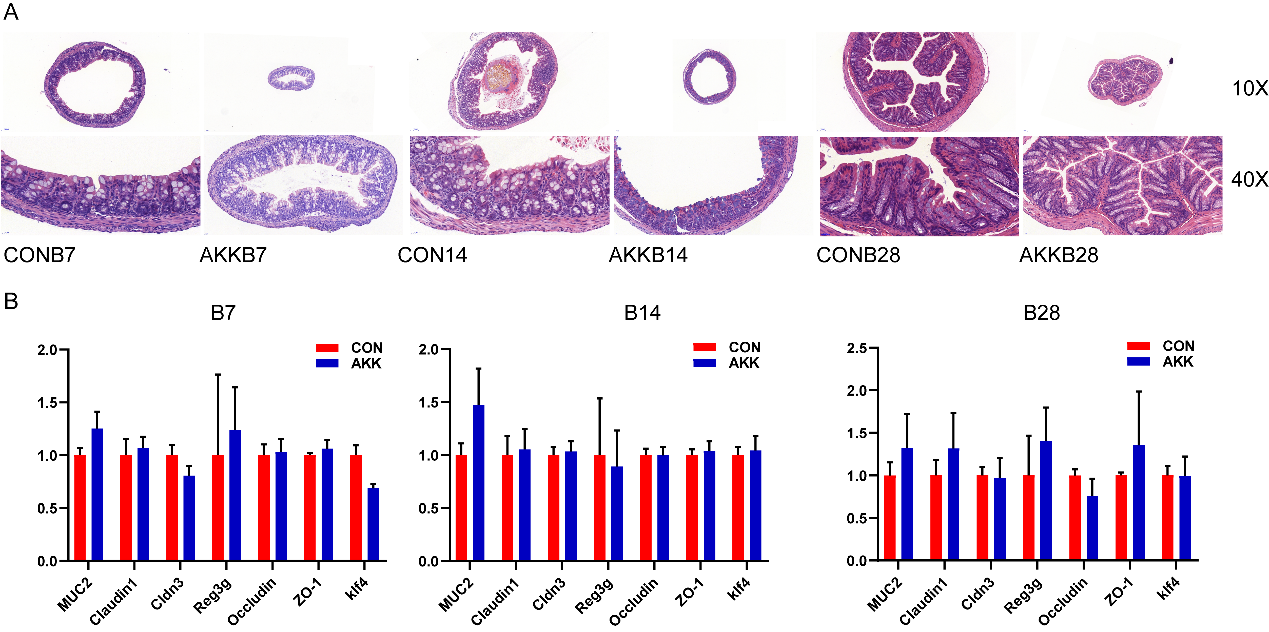


**Figure S2.** Effects of *A. muciniphila* supplementation during pregnancy and lactation on the intestinal barrier of mouse offspring. (A) Representative image of hematoxylin-eosin-stained colon tissue of offspring at different points after birth. (B) Comparison of mRNA expression of genes related to the intestinal barrier of mouse offspring.


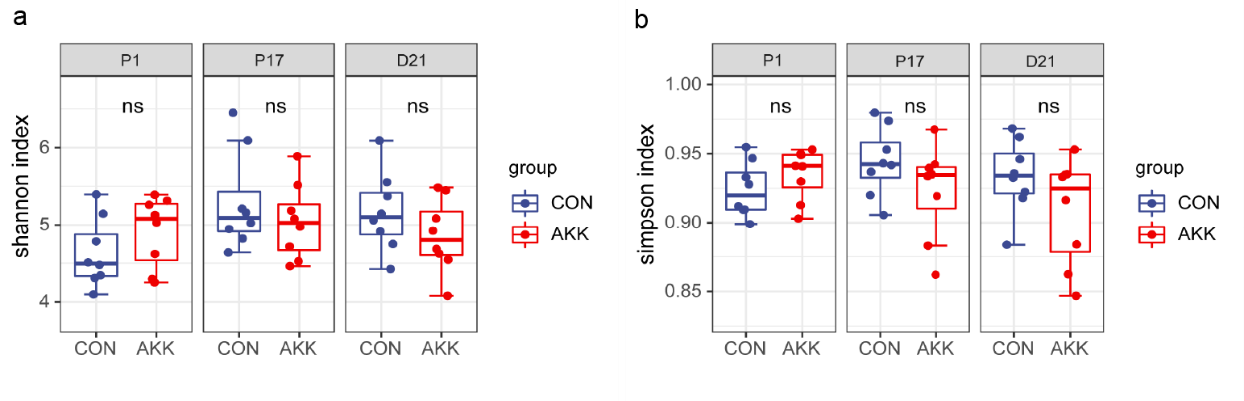


**Figure S3.** *A. muciniphila* supplementation results in favorable alterations in maternal gut microbiota. (a) The shannon index of mother mice. (b) The simpson index of mother mice.


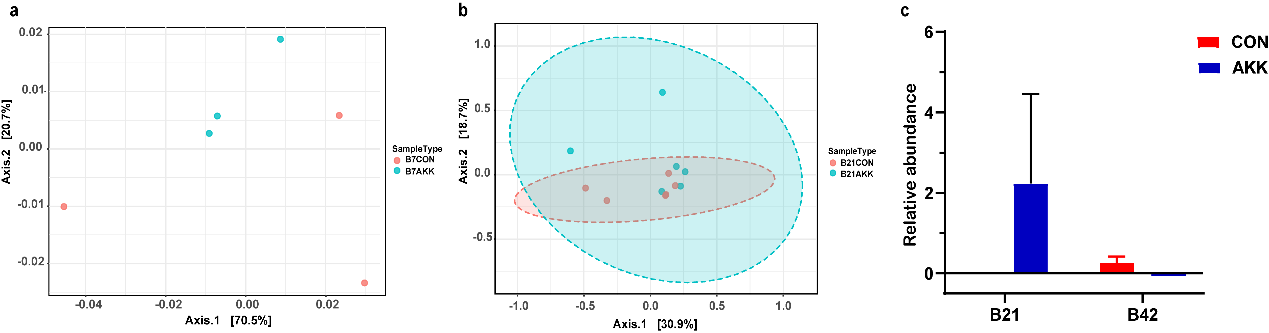


**Figure S4.** *A. muciniphila* supplementation results in favorable alterations in the gut microbiota of pups. PCoA describing the β-diversity clustering of the gut microbiota of pups at B7 (a) and B21 (b). (c) The relative abundance of *A. muciniphila* concentration in offspring feces.
